# Lipid-Nanoparticle-Mediated Delivery of Docetaxel Prodrug for Exploiting Full Potential of Gold Nanoparticles in the Treatment of Pancreatic Cancer

**DOI:** 10.3390/cancers14246137

**Published:** 2022-12-13

**Authors:** Abdulaziz Alhussan, Nolan Jackson, Sarah Eaton, Nancy Dos Santos, Ingrid Barta, Josh Zaifman, Sam Chen, Yuen Yi C. Tam, Sunil Krishnan, Devika B. Chithrani

**Affiliations:** 1 Department of Physics and Astronomy, University of Victoria, Victoria, BC V8P 5C2, Canada; 2 Department of Experimental Therapeutics, British Columbia Cancer-Vancouver, Vancouver, BC V5Z IL3, Canada; 3 Centre for Comparative Medicine, Animal Care Services, University of British Columbia, Vancouver, BC V6T 1W5, Canada; 4 Integrated Nanotherapeutics Inc., Burnaby, BC V5G 4X4, Canada; 5 Neurosciences, The University of Texas Health Science Center, Houston, TX 77030, USA; 6 Radiation Oncology, British Columbia Cancer-Victoria, Victoria, BC V8R 6V5, Canada; 7 Centre for Advanced Materials and Related Technologies, Department of Chemistry, University of Victoria, Victoria, BC V8P 5C2, Canada; 8 Centre for Biomedical Research, Department of Biology, University of Victoria, Victoria, BC V8P 5C2, Canada; 9 Division of Medical Sciences, University of Victoria, Victoria, BC V8P 5C2, Canada; 10 Department of Computer Science, Mathematics, Physics and Statistics, Okanagan Campus, University of British Columbia, Kelowna, BC V1V 1V7, Canada

**Keywords:** gold nanoparticles, docetaxel, lipid nanoparticles, pancreatic cancer, nanomedicine

## Abstract

**Simple Summary:**

Pancreatic cancer is one of the leading causes of cancer deaths worldwide. The use of nanoparticles as radiosensitizers and drug delivery vehicles could open the door to solving many of the obstacles in current cancer treatments. Gold nanoparticles (GNPs) and docetaxel (DTX) have shown very promising synergetic radiosensitization effects, despite DTX toxicity to normal tissues. In this paper, we explored the effect of a DTX prodrug encapsulated in lipid nanoparticles (LNP_DTX-P_) on GNP uptake in pancreatic cancer models in vitro and in vivo. The results show that LNP_DTX-P_-treated tumour samples have twice the amount of GNP uptake in both in vitro and in vivo models. These very promising results establish that LNP_DTX-P_ have very similar outcomes to free DTX on tumour tissues. These results demonstrate the potential of incorporating GNPs and LNP_DTX-P_ as radiosensitization tools to current radiotherapy protocols for improved tumour targeting.

**Abstract:**

Current chemoradiation therapy suffers from normal tissue toxicity. Thus, we are proposing incorporating gold nanoparticles (GNPs) and docetaxel (DTX), as they have shown very promising synergetic radiosensitization effects. Here, we explored the effect of a DTX prodrug encapsulated in lipid nanoparticles (LNP_DTX-P_) on GNP uptake in pancreatic cancer models in vitro and in vivo. For the in vitro experiment, a pancreatic cancer cell line, MIA PaCa-2, was cultured and dosed with 1 nM GNPs and 45 nM free DTX or an equivalent dose of LNP_DTX-P_. For the in vivo experiment, MIA PaCa-2 cells were implanted subcutaneously in NRG mice, and the mice were dosed with 2 mg/kg of GNPs and 6 mg/kg of DTX or an equivalent dose of LNP_DTX-P_. The results show that LNP_DTX-P_-treated tumour samples had double the amount GNPs compared to control samples, both in vitro and in vivo. The results are very promising, as LNP_DTX-P_ have superior targeting of tumour tissues compared to free DTX due to their nanosize and their ability to be functionalized. Because of their minimal toxicity to normal tissues, both GNPs and LNP_DTX-P_ could be ideal radiosensitization candidates in radiotherapy and would produce very promising synergistic therapeutic outcomes.

## 1. Introduction

The National Cancer Institute declares pancreatic ductal adenocarcinoma (PDAC) the third leading cause of cancer-related deaths in North America. PDAC has one of the lowest survival rates of all major cancers, with a 5-year survival rate of lower than 8% [1]. The invasiveness and metastatic nature of PDAC make the majority of cases not fit for surgery [2,3]. A key component of the treatment of non-metastatic PDAC is chemoradiation therapy, where the addition of radiosensitizing chemotherapy to radiotherapy (RT) has marginally improved survival [4,5]. Nonetheless, most patients surrender to their illness within the first year [6]. The currently used free-form chemotherapeutic drugs suffer from a major dilemma wherein only a small amount of the injected drug reaches the tumour, with no appreciable variation in drug concentrations between the tumour and healthy organs [7,8]. Normal tissue toxicity limits the delivery of safe doses of radiation and chemotherapeutic drugs without complications to vital organs. Hence, exploring alternative approaches necessitates further examination. Nanotechnology offers a practical solution to many of these challenges.

High-atomic-number metallic nanoparticles (NPs), such as gold nanoparticles (GNPs), have shown encouraging effects as radiosensitizing agents in various preclinical models of cancer [9]. GNPs can be functionalized with polyethylene glycol (PEG) and the integrin-binding peptide RGD. The former works as a protective coating that prevents protein adsorption on the surface of the nanoparticle (opsonization) and clearance by the reticuloendothelial system, thereby allowing for longer circulation time [9]. The latter is used due to the high expression of integrin dimers on pancreatic cancer cells, which can recognize the RGD motif, allowing for improved cancer cell targeting [10]. On the other hand, lipid-based nanoparticles (LNPs) can be utilized as drug delivery vectors and functionalized to specifically target tumours with controlled delivery, thus significantly decreasing the dose delivered to healthy organs [11]. Additionally, LNPs’ long circulation time results in an increase of up to 2–5% of the injected dose within the tumour compared to 0.1% of the free-form drug, which has a short half-life [12]. Assuming only 1% of the LNP drug reaches the target site, this still results in over 10 times the intratumoural concentration of the drug compared to the free-form drug [13]. In addition to these advantages, the small size of the NPs allows them to reach the tumour via the enhanced permeability and retention (EPR) effect due to the leaky tumour vasculatures [7].

The combination of GNPs and low concentrations of docetaxel (DTX) have been shown to radiosensitize cancer cells in vitro [14]. GNPs can radiosensitize cancer cells by increasing the production of free radicals [15]. On the other hand, DTX impedes the cell division cycle and traps cells in the most radiosensitive phases of mitosis, the G2/M phases [16]. Because of this, DTX has been used as a radiosensitizer in several clinical trials [17,18]. Moreover, DTX damages cell microtubules (MTs), blocking cell division, which leads to a significant accumulation of GNPs within cells over time [19]. Hence, the coupling of these two radiosensitizers with RT could result in a synergistic therapeutic outcome. The problem is that the effectiveness of free DTX is considerably compromised due to its toxicity to normal tissues and poor solubility [20]. To overcome these issues, we have developed a DTX prodrug encapsulated in LNPs (LNP_DTX-P_).

**Figure 1:**
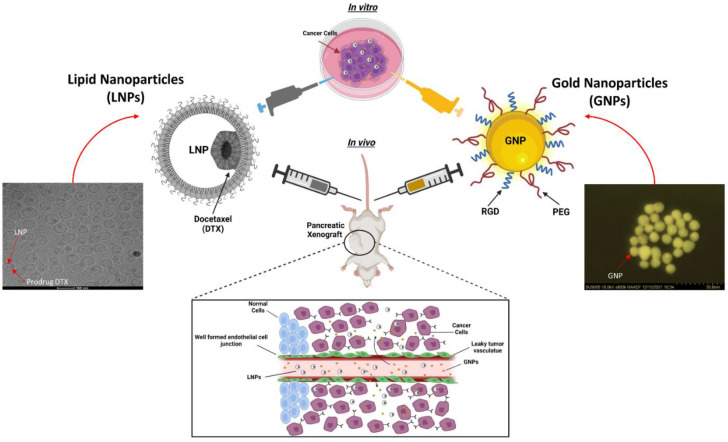
Schematic showing preclinical xenograft model to test the efficacy of a docetaxel (DTX) prodrug encapsulated in a lipid nanoparticle (LNP) and gold nanoparticle (GNP) combination vs. a 2D in vitro model with a high-angle annular darkfield (HAADF) image of GNP and a cryogenic transmission electron microscopy image of LNP_DTX-P_. The inset shows a schematic diagram of the escape of NPs from leaky blood vessels to tumour tissues.

In this study, we assessed the biodistribution of GNPs in the presence of LNP_DTX-P_ vs. free DTX in MIA PaCa-2 pancreatic-carcinoma-bearing NRG mice and compared it to the 2D monoculture model of MIA PaCa-2 cells (Figure 1). GNPs were functionalized with PEG and RGD, and two different DTX prodrug concentrations were used in the LNP_DTX-P_, a 5% (by weight) concentration referred to as LNP_DTX-1_ and a 10% (by weight) concentration referred to as LNP_DTX-2._ This unique combination of GNPs and LNP_DTX-P_ has not been explored in vitro or in vivo, which would allow us to find the optimal time point where cancer cells have the most gold in them and are trapped in the radiosensitive G2/M phases. This step is essential before proceeding to the combined GNP+LNP_DTX-P_+RT treatment, which will be explored in future experiments.

## 2. Materials and Methods

### 2.1. Gold Nanoparticle Synthesis, Functionalization, and Characterization

The citrate reduction method was used to make gold nanoparticles (GNPs) of sizes around 11 nm in diameter. First, 2.36 mL of 1% tetrachloroauric (III) acid trihydrate (1015820001; Sigma-Aldrich, St. Louis, MO, USA) was mixed with 57.64 mL of water and heated. Once boiled, 2.4 mL of 5% sodium citrate tribasic dihydrate (S4641; Sigma-Aldrich, St. Louis, MO, USA) was added and left for 10 min until the solution color shifted to red. Then, the solution was stirred at 20 °C for 15 min. For functionalization, 2000 Da polyethylene glycol (PEG) (B2010146, Molecular Depot, San Diego, CA, USA) and 1600 Da arginine–glycine–aspartic acid (RGD) (AnaSpec, San Jose, CA, USA) were used at a surface density of 1 PEG per nm^2^ of GNP surface area and 1 RGD for every 2 PEG. For confocal imaging, PEG-thiol-CY5 (PG2-S5TH-2k; Nanocs, Boston, MA, USA) was added to the GNP. A Perkin Elmer λ 365 ultraviolet-visible (UV-VIS) spectrophotometer (Waltham, MA, USA) was used to approximate the size and concentration of the NPs. The surface charge and hydrodynamic diameter were determined using the ζ potential and dynamic light scattering (DLS) (Anton Paar LiteSizer 500, Graz, Austria). The shape and size of the NPs were validated using high-angle annular darkfield (HAADF) scanning transmission electron microscopy (STEM) (SU9000 Ultra-high Resolution Scanning Electron Microscope, Hitachi, Pleasanton, CA, USA).

### 2.2. Lipid Nanoparticle Synthesis and Characterization

LNPs were prepared by rapid mixing [21]. Briefly, cholesterol (C8667; Sigma–Aldrich, St. Louis, MO, USA), DSPC (850365C; Avanti Polar Lipids, AL, Alabama, USA), PEG-DSPE (880128C; Avanti Polar Lipids, Alabaster, AL, USA), and DTX prodrugs (refer to the supplementary section for the synthesis of DTX prodrugs, Figure S1) were dissolved in ethanol to a final concentration of 10 mM and a molar ratio of 40:49:1:10, respectively. For formulations that contained less than 10 mol% DTX prodrug, DSPC and cholesterol were increased at a fixed molar ratio. The lipids in ethanol were mixed with phosphate-buffered saline (PBS, pH 7.4) at a flow ratio of 1:4 (*v*:*v*) and a total flow rate of 40 mL/min. The resulting mixtures were then dialyzed in 1000-fold volumes of PBS overnight and sterile-filtered (0.2 µm). The particle size was determined by DLS using a Malvern Zetasizer NanoZS (Malvern Instruments, Worcestershire, UK). Lipid concentrations were determined by measuring the cholesterol and phospholipid contents of the LNPs (Cholesterol E Assay or Phospholipids C Assay, Wako Chemicals, Richmond, VA, USA). Ultra-performance liquid chromatography (UPLC) (Waters, Milford, Massachusetts, United States) was used to determine the final concentrations of the DTX prodrug. A Waters Acquity H-Class UPLC System equipped with a BEH C18 column (1.7 µm, 2.1 mm × 100 mm) and a photodiode array detector was used. Separation was achieved at a flow rate of 0.5 mL/min with a linear gradient of mobile phases of acetonitrile to water from 20:80 to 100:0 (*v*:*v*) over 3 min followed by an isocratic hold at 100:0 for an additional 3 min. The column temperature was maintained at 55 °C. The absorbance at 230 nm was measured, and the analyte concentration was determined using calibration curves. Prodrug entrapment was determined by comparing the prodrug-to-cholesterol ratios in the final LNPs to that of the initial lipid mixtures. Cryogenic transmission electron microscopy was performed by the High-Resolution Macromolecular Cryo-Electron Microscopy Facility at The University of British Columbia (Vancouver, BC, Canada).

### 2.3. Cell Culture

The human pancreatic cancer cell line MIA PaCa-2 (ATCC#: CRL-1420™) was used. For both in vitro and in vivo experiments, cell cultures of passages 3 to 10 and a confluence of 70–90% were used. For in vitro experiments, cultured cells were supplied with high-glucose Dulbecco’s Modified Eagle’s Medium (DMEM, 11965092; Gibco, ThermoFisher Scientific, Waltham, MA, USA) enhanced with 10% fetal bovine serum (FBS, A5256701; Gibco, ThermoFisher Scientific, Waltham, MA, USA) and 4 mM GlutaMax (35050079; Gibco, ThermoFisher Scientific, Waltham, MA, USA). For in vivo experiments, cells were cultured in Ham’s F12 medium (21127030; Gibco, ThermoFisher Scientific, Waltham, MA, USA) enhanced with 2 mM L-glutamine (A2916801; Gibco, ThermoFisher Scientific, Waltham, MA, USA) and 10% FBS (Gibco, ThermoFisher Scientific, Waltham, MA, USA). For cell detachment from flasks, TrpyLE (12605010; Gibco, ThermoFisher Scientific, Waltham, MA, USA) was used. For cell fixation, paraformaldehyde (PFA, 047392.9M; Sigma-Aldrich, St. Louis, MO, USA) was used. For cell washing, phosphate-buffered saline (PBS, J61196.AP, ThermoFisher Scientific, Waltham, MA, USA) was used. All cell incubations were at 37 °C with 5% CO_2_.

### 2.4. Proliferation Assay

A proliferation assay was used to determine the IC-50 of DTX (D543642; eNovation Chemicals, Bridgewater, NJ, USA), LNP_DTX-1_ (refer to Section 2.2; Integrated Nanotherapeutics; Burnaby, BC, Canada), and LNP_DTX-2_ (refer to Section 2.2; Integrated Nanotherapeutics; Burnaby, BC, Canada). For each drug, 3 black-walled clear-bottom 96-well plates (Greiner, Monroe, NC, USA) were used. About 10^4^ of the cells were seeded in each plate with 100 μL of fresh medium, with one column left unseeded and covered with a breathable membrane (Breathe-Easier Membranes; Sigma-Aldrich; St. Louis, MO, USA). For all three drugs used, a maximum starting dose of 9000 nM was used in one column, and concentrations were serially diluted by thirds in each consecutive column. The membrane was disposed of 24 h post-treatment, and the medium was changed. Cell viability was measured 48 h postdosing using a medium containing 10% *v*/*v* resazurin dye PrestoBlue (A13261; Thermo-Fisher, Waltham, MA, USA) after 30 min of incubation. Fluorescence was measured using a Biotek Cytation 1 plate reader (filters at excitation of 530/25 nm and emission of 590/35 nm, Winooski, VT, USA).

### 2.5. Xenograft Model

For the in vivo experiments, 6–10-week-old male NRG mice were purchased from the BC Cancer Research Institute Animal Resource Centre (ARC, Vancouver, BC, Canada). Mice were caged in autoclaved Allentown ventilated caging at a capacity of 2–4 animals/cage for the length of the study. Cages were changed biweekly and included Nestlets (Ancare, Bellmore, NY, USA) as environmental enrichment, transparent tinted polycarbonate Mouse Igloos (Bio-Serv, Flemington, NJ, USA), and Envigo 7097 ¼” corn cob bedding. All enrichment was added to the cages prior to the cages being autoclaved. Mice were fed Envigo Teklad Global Rodent Diet 2920 (Indianapolis, IN, USA). The rodent food was kept in the hoppers of the wire lids and was changed biweekly. Reverse-osmosis water was supplied through Avidity Science automatic watering valves at a flow rate of 25–50 mL/min. The environmental control of the lights and the monitoring of temperature, humidity, and airflow was performed by WatchDogEX™ (Waterford, WI, USA). On the first day of the experiment, 5 × 10^6^ tumour cells were implanted subcutaneously into each mouse’s back. The volumes of the tumours were determined using the following formula: L *×* W^2^ × 0.5.

### 2.6. Treatment of Xenograft Model

The mice were randomly assigned to 5 study groups: A. untreated, B. GNP_PEG–RGD_ only, C. GNP_PEG–RGD_ and LNP_DTX-1_, D. GNP_PEG–RGD_ and LNP_DTX-2_, and E. GNP_PEG–RGD_ and free DTX. For group A., 6 mice were allocated to measure the MIA PaCa-2 tumour growth kinetics. For the remaining groups (B–E), 12 mice were allocated to each group, with 4 mice per three different time points: 8, 24, and 48, hours after dosing. The treatment of mice to assess the pharmacokinetics in the blood and the tissue biodistribution began when the tumours were 250–300 mm^3^. GNPs and the drugs were administered concurrently and intravenously. GNP_PEG–RGD_ were dosed at 2 mg/kg of mouse. DTX (Sandoz) was supplied in a 10 mg/mL solution that had 96% citric acid, ethanol, PEG 300, and polysorbate 80. The dose for DTX was 6 mg/kg of mouse. Both LNP_DTX-1_ and LNP_DTX-2_ were provided in 4.5 mg/mL PBS and dosed at 6 mg/kg of mouse.

### 2.7. Pharmacokinetic Tissue Sampling

Mice were individually weighed and injected with the treatments listed in Section 2.6 according to the study group, and blood was sampled. Once at the experimental endpoint, mice were euthanized according to an approved animal care protocol, terminal blood was collected by cardiac puncture, and tissues were harvested. For hematology, whole blood from the endpoint cardiac puncture of 1 of 4 mice was placed into a K2 EDTA tube, gently inverted a minimum of 8–10 times to ensure no clotting occurred, and then placed on ice. Samples were sent to IDEXX for a complete blood count (CBC) analysis after collection. For each time point, 50% of the tumour from 2 out of 4 mice was collected into 10% neutral buffered formalin for histopathology, while all mice had 50% of their tumours frozen for use in the biodistribution study. The entire tumours of 2 out of 4 mice from each time point were placed in 70% ethanol for further cell cycle analysis. Similarly, 1 out of 4 mice at each time point was used for organ histopathology, where the liver, spleen, and kidneys were placed in 10% neutral buffered formalin, and 3 out of 4 mice had these organs and blood plasma frozen for use in the biodistribution study.

### 2.8. Histopathology

First, 10% neutral buffered formalin-fixed tissues were processed into paraffin overnight using an automated tissue processor, embedded, and sectioned at 4 µm. Two slides were collected from each of the two levels spaced 50 µm apart. Half of the slides were simply prepared with a resinous mounting medium for darkfield imaging, and the other half were stained with hematoxylin and eosin (H&E) according to standard procedures for brightfield imaging. A darkfield (DF) coupled with hyperspectral imaging (HSI) CytoViva microscope (CytoViva, Auburn, AL, USA) was used to determine GNP localization within cells.

### 2.9. Cellular Uptake of Gold Nanoparticles

For the in vitro experiment, 1 × 10^5^ cells were seeded and incubated for 24 h in 6-well dishes with 3 mL of medium. For each treatment condition and each time point, 3 wells were used. After 24 h, cells were concurrently dosed with 1 nM GNP_PEG−RGD_ and the IC-50 dose of DTX or an equivalent DTX dose of LNPs. Cells were then incubated for 24 h. After the 24 h incubation period, the uptake plates were ready for processing, while the media of the 24 h and 48 h retention plates were changed and cells were further incubated for 24 h and 48 h, respectively. To process the cells, cells were washed 3 times with PBS, trypsinized, and incubated for 5 min. The medium was then added to the cells, and they were counted using a hemocytometer counting chamber and transferred to glass tubes for processing. Cells were then treated with aqua regia and heated in a mineral oil bath at 90 °C for 30 min. For each tube, 100 μL of hydrogen peroxide was added, followed by incubation in a mineral oil bath for another 30 min. The samples were then diluted in deionized water. For the in vivo samples, the samples were weighed, blended with 2 mL of TrypLE, and left to break down. The samples were then diluted in Millipore water and treated with 250 μL of aqua regia per 500 μL of each sample in a 90 °C mineral oil bath for a minimum of 2 h. The samples were then diluted to 2.5% in deionized water before being filtered with a 0.2-micron filter (Waltham, MA, USA). Inductively coupled plasma–mass spectrometry (ICP-MS; Agilent 8800 Triple Quadrupole, Agilent Technologies, Santa Clara, CA, USA) was utilized to quantify the amount of gold in the samples.

### 2.10. Preparation of Cells for Imaging

Approximately 5 × 10^4^ cells were seeded in 35 mm coverslip-bottom dishes (MatTek, Ashland, MA, USA) with 2 mL of medium and were incubated for 24 h. One day after seeding, the cells were concurrently dosed with GNP_PEG-CY5-RGD_ at 1 nM and the IC-50 dose of either DTX, LNP_DTX-1_, or LNP_DTX-2_. Then, 16 h prior to imaging, the tubulin stain CellLight™ Tubulin-GFP (C10613; BacMam 2.0, ThermoFisher Scientific, Waltham, MA, USA) was added. Next, 24 h post-treatment, 4 drops of the live reagent (DAPI) NucBlue^®^ (R37605; ThermoFisher Scientific, Waltham, MA, USA) was added per dish, followed by incubation for 20 min. Live cell imaging was performed using a 60X oil-immersion lens for confocal microscopy (Zeiss LSM 980, Carl Zeiss Microscopy GmbH, Jena, Germany).

### 2.11. Cell Cycle Analysis

For the in vitro experiment, cells were cultured in 60 mm dishes with 5 mL of medium and were incubated for 24 h. Cells were then treated with the IC-50 dose of DTX or the equivalent DTX dose of LNPs. After their corresponding incubation times, cells were trypsinized and neutralized in medium. For the in vivo experiment, the samples were treated with Collagenase/Dispase (Roche 10269638001; Sigma Aldrich, St. Louis, MO, USA) for two hours. The samples were then filtered through a 100-micron cell strainer and were treated along with the in vitro samples, as described next. All samples were centrifuged at 350× *g* for 5 min at 4 °C. The cell pellets were then washed with PBS and centrifuged again at 350× *g* for 5 min at 4 °C. The cells were then fixed with PFA and incubated in the fridge for 15 min. The samples were then centrifuged at 350× *g* for 5 min at 4 °C, washed with PBS, and centrifuged again at 350× *g* for 5 min at 4 °C before being resuspended in 70% ethanol and incubated at −20 °C for 2 days. After that, the samples were centrifuged at 350× *g* for 10 min at 20 °C, washed with 0.5% bovine serum albumin (BSA, A1933; Sigma Aldrich, St. Louis, MO, USA) in PBS, and then centrifuged at 350× *g* for 5 min at 20 °C. Following that, the samples were incubated on a shaker with PBTB (PBS, 0.5% BSA, 0.1% Triton-X 100) and RNaseA (10109142001; Sigma Aldrich, St. Louis, MO, USA) at 37 °C for 25 min. Then, propidium iodide (P4170; ThermoFisher Scientific, Waltham, MA. USA) was added to the samples and incubated on a shaker at 4 °C for 60 min. After that, the samples were centrifuged at 350× *g* for 10 min at 20 °C. Finally, the samples were resuspended in PBS/BSA and filtered using a 50 µm cell strainer. The samples were run on a flow cytometer (FACS Calibur, BD Biosciences, Franklin Lakes, NJ, USA).

### 2.12. Statistical Analysis

Welch’s *t*-test in python was performed for the statistical analysis. The experiments were repeated 3 times, and the error bars indicate one standard deviation.

## 3. Results and Discussion

### 3.1. Docetaxel Prodrug Lipid Nanoparticle Effects In Vitro

**Figure 2:**
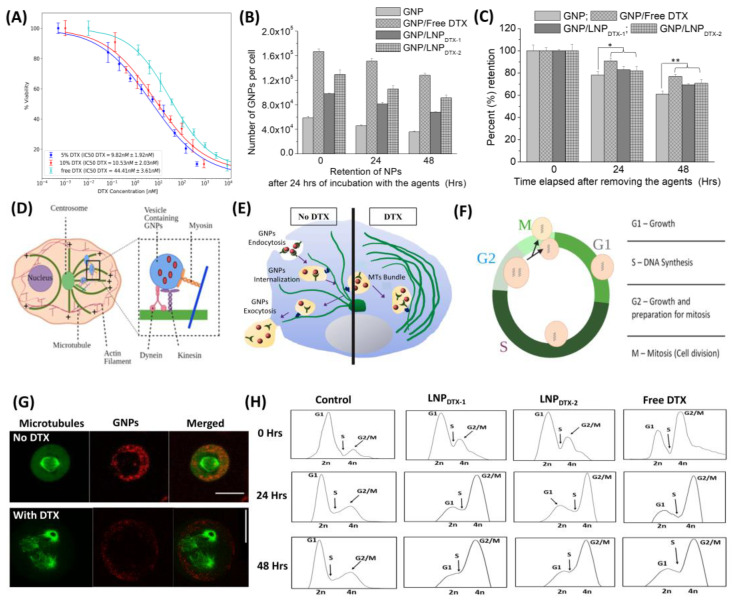
Effect of free docetaxel (DTX) vs. LNP_DTX-P_ on MIA PaCa-2 cells in vitro. (**A**) IC-50 curves of free DTX, 5% DTX (LNP_DTX-1_), and 10% DTX (LNP_DTX-2_). (**B**) Amounts of gold nanoparticles (GNPs) per cell in control cells, cells treated with free DTX, cells treated with LNP_DTX-1_, and cells treated with LNP_DTX-2_ over time. (**C**) GNP retention over time for control cells, cells treated with free DTX, cells treated with LNP_DTX-1_, and cells treated with LNP_DTX-2_. * indicates *p* < 0.05, ** indicates *p* < 0.01. (**D**) Visual illustration of GNP movement within a cell’s MTs [19]. (**E**) Schematic diagram illustrating the path of GNPs (red dots) within a cell in the absence and presence of DTX. (**F**) Cell cycle phases. In preparation for cell division, the cell goes through three different phases: a G1 gap phase between the M and S phases, an S phase where DNA replication occurs, and G2 where the cell prepares for mitosis. (**G**) Confocal images of dividing cancer cells: control (top) and DTX (bottom). GNPs are shown in red, and microtubules are shown in green. The scale bar: 25 µm. (**H**) Cell cycle assay for control cells, cells treated with free DTX, cells treated with LNP_DTX-1_, and cells treated with LNP_DTX-2_ over time.

To measure the toxicity effect of free DTX and the two LNP_DTX-P_, LNP_DTX-1_ and LNP_DTX-2_, on MIA PaCa-2 cells, a proliferation assay was used. Based on this assay, the half-maximal inhibitory concentrations (IC-50s) of free DTX, LNP_DTX-1_, and LNP_DTX-2_, on MIA PaCa-2 were determined to be 44.41 ± 3.61 nM, 9.82 ± 1.92 nM, and 10.53 ± 2.03 nM, respectively (Figure 2A). These results are very promising, as they show that delivery of DTX in LNP_DTX-P_ reduces the IC-50 by approximately four-fold. We attribute the enhanced efficacy to the use of prodrugs in the LNPs. The DTX prodrug was designed to be stably incorporated into the LNPs and consists of DTX conjugated to a hydrophobic anchor by a biodegradable linker [21]. Within the LNP, the prodrug resides within a hydrophobic pocket due to its poor water solubility. Following the LNP_DTX-P_ uptake by the cells, the DTX prodrug undergoes biotransformation. The intracellular enzymes break down the biodegradable (ester) linker, releasing the active DTX [21]. Consequently, LNP_DTX-P_ increased the ability of transport, improved tumour targeting, enhanced therapeutic effectiveness, and minimized drug-induced toxicity in normal tissues [21,22,23,24,25,26]. These LNP platforms have been used successfully in the clinic to deliver nucleic acids and small-molecule chemotherapeutics [27,28,29]. Therefore, clinically relevant intratumoural concentrations of DTX can be achieved by formulating a DTX prodrug into LNPs.

The characterization of GNPs and LNPs is displayed in Figure S2, which displays the successful conjugation of PEG and RGD into the GNPs and the stability of the GNP/LNP mixed solution. This was indicated by the increases in the hydrodynamic diameter and the zeta potential charge of the GNPs following the conjugation and the stability of the zeta potential charge following the mixing of the two solutions. To compare the efficiency of free DTX and LNP_DTX-P_ in vitro, we measured the number of GNPs at three different time points after being dosed with GNP_PEG-RGD_ at a clinically relevant concentration of 1 nM and treated with the IC-50 dose of free DTX or the equivalent dose of LNP_DTX-P_ (Figure 2B). The results clearly show a higher number of GNPs in the cells treated with DTX or LNP_DTX-P_ compared to the control cells. The numbers of GNPs in the DTX-treated cells, LNP_DTX-1_-treated cells, and LNP_DTX-2_-treated cells were 2.8 times, 1.7 times, and 2.2 times higher than in the control cells for the 0 h time point; 3.3 times, 1.8 times, and 2.3 times higher for the 24 h time point; and 3.6 times, 1.9 times, and 2.5 times higher for the 48 h time point, respectively. Figure 2C shows the retention of GNPs for control cells to be 77% and 61% of their initial GNPs after 24 h and 48 h, respectively. These retention percentages significantly increased for the LNP_DTX-1_-treated cells to 82% and 71%, for the LNP_DTX-2_-treated cells to 83% and 70%, and for the free-DTX-treated cells to 91% and 77%, respectively. These results signify that free DTX and LNP_DTX-P_ not only significantly increased the number of GNPs in cells compared to the control cells but also significantly increased their retention.

These results are better explained when understanding the effect of DTX on cell microtubules (MTs) and on GNP cellular transportation. GNPs are internalized via receptor-mediated endocytosis (RME), where cell surface receptors bind to the RGD ligand on the NPs surface and become engulfed by endosomes [30,31,32]. The NPs are then pulled along MTs by two molecular motors, kinesin and dynein, and along actin by the motor myosin (inset in Figure 2D) [19]. After that, the NPs are fused with lysosomes to be sorted out, where any waste is excreted out of the cell (Figure 2E left) [32]. MTs play a major role in cell division and facilitate the movement of GNPs inside cells. Therefore, the interference in MT function caused by DTX can substantially affect the GNP intracellular journey (Figure 2E, right) [33,34]. One of the key mechanisms of action of the FDA-approved drug DTX is the inhibition of MT depolymerization, which leads to defective MT bundles, impeding the proper development of spindle apparatuses, which are involved in mitosis [35,36,37,38]. In a normal M phase (Figure 2F), MTs and the microtubule-organizing centre (MTOC) are used to create mitotic spindles that then equally pull the chromosomes into the divided cells (Figure 2G, top). However, with doses of just 50 nM DTX, MTs malfunctioned, and cells became locked in mitosis (Figure 2G, bottom) [39]. Over time, this resulted in arresting the cell cycle at the G2/M phases, the most radiosensitive phases of the cell cycle for both the free DTX and the LNP_DTX-P_, as explained by the flow cytometry data (Figure 2H). Although initially LNP_DTX-P_ had a slower shift from the G1 phase cell population to the G2 phase cell population compared to free DTX, they eventually had similar cell synchronizations after approximately 24 h. This led to GNPs becoming trapped within the cell because of their inability to move along the damaged MTs, resulting in a deficiency in the cell’s ability to secrete GNPs. Hence, it not only increased GNP uptake into cells but also blocked their exit by not allowing cells to divide and distribute GNPs into the daughter cells. This explains the increases in both uptake and retention in free-DTX- and LNP_DTX-P_-treated cells.

**Figure 3:**
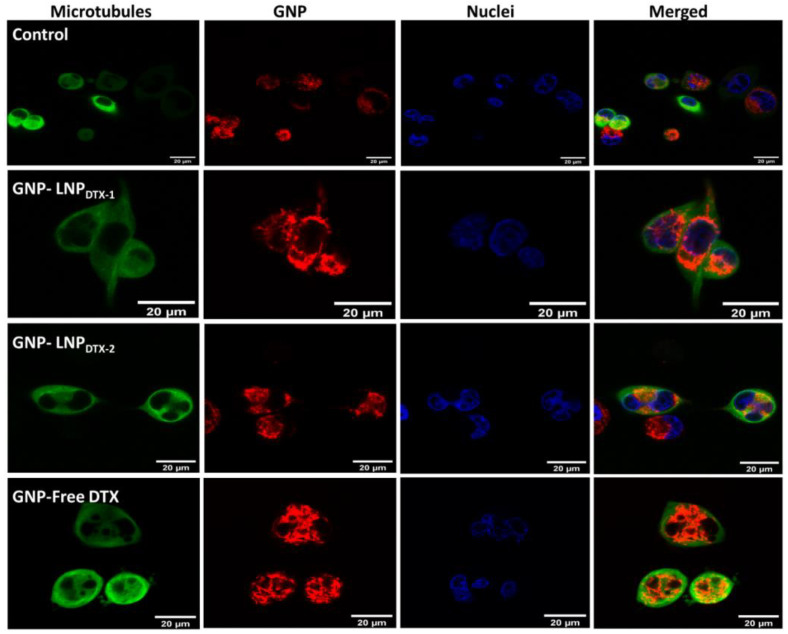
Visualization of intracellular GNP distribution in MIA PaCa-2 cells using confocal imaging. Control untreated cells (1st row), cells treated with LNP_DTX-1_ (2nd row), cells treated with LNP_DTX-2_ (3rd row), and cells treated with free DTX (4th row). Microtubules in green (1st column), GNPs in red (2nd column), nuclei in blue (3rd column), and all three merged (4th column). Scale bar: 20 µm.

Confocal images of cancer cells treated with free DTX, LNP_DTX-1_, and LNP_DTX-2_ vs. control cells show some multinucleated cells caused by this mechanism of action of DTX (Figure 3). As explained earlier, these treated cells were unable to divide properly and were trapped in the M phase, where some developed multinucleation. These results are consistent with other studies that reported significant increases in GNP uptake with the treatment of DTX in multiple cell lines [19,39].

### 3.2. Docetaxel Prodrug Lipid Nanoparticle Effects In Vivo

**Figure 4:**
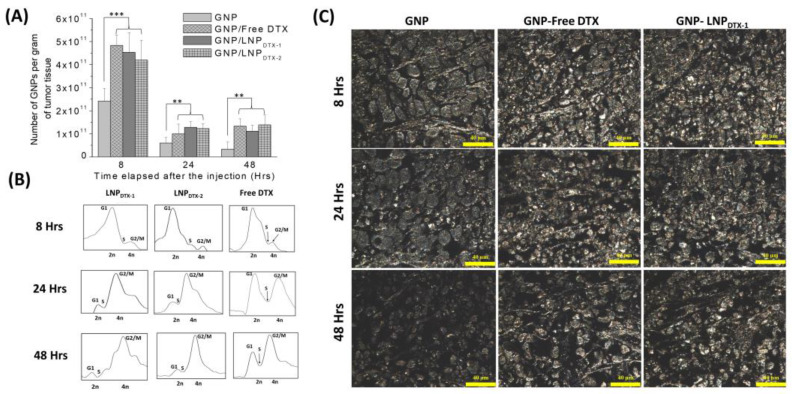

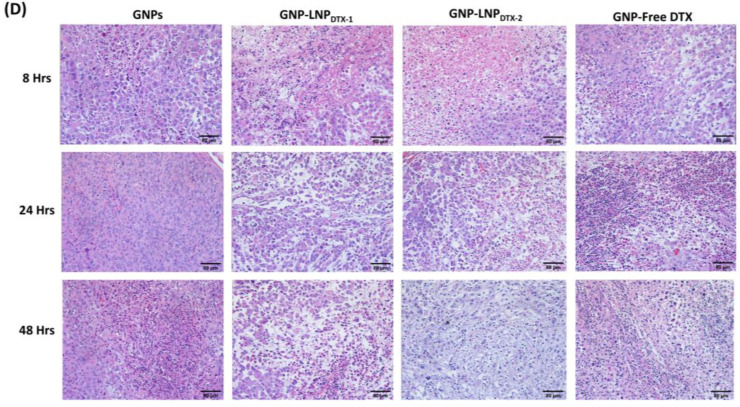
Effects of docetaxel (DTX) vs. LNP_DTX-P_ on in vivo tumour tissues. (**A**) Amounts of gold nanoparticles (GNPs) per gram of tumour tissue in untreated tissues, tissues treated with free DTX, tissues treated with LNP_DTX-1_, and tissues treated with LNP_DTX-2_ over time. ** indicates *p* < 0.01, *** indicates *p* < 0.001. (**B**) Cell cycle assay for untreated tumour tissue, tissue treated with free DTX, tissue treated with LNP_DTX-1_, and tissue treated with LNP_DTX-2_ over time. (**C**) Darkfield images of 4 µm sections of untreated tumour tissues, tissues treated with free DTX, and tissues treated with LNP_DTX-1_. Scale bar: 40 µm. (**D**) Treatment of MIA PaCa-2 subcutaneous tumour with free docetaxel (DTX) vs. LNP_DTX-P_. Hematoxylin and eosin stained sections of tumour tissues 0 h, 24 h, and 48 h after dosing with the drugs and GNPs. Scale bar: 80 µm.

In this experiment, MIA PaCa-2 cells were implanted subcutaneously in NRG mice and then dosed intravenously with GNPs and either free DTX or LNP_DTX-P_ to assess the drug toxicity and biodistribution over time once tumours reached a measured volume of 250–300 mm^3^. The free DTX dose used was 6 mg/kg or an equivalent dose of LNP_DTX-P_, and GNP_PEG–RGD_ were dosed at 2 mg/kg. These doses were shown to be tolerable for in vivo administration, with a goal in mind for future clinical applications [40,41,42,43]. GNP uptake per tumour tissue revealed that free-DTX- and LNP_DTX-P_-treated mice had twice the number of GNPs in their tumours compared to the control mice (Figure 4A). The leaky vasculatures and ineffective lymphatic systems at tumours facilitate the accumulation of NPs in tumour cells in a process known as the enhanced permeability and retention (EPR) effect [7]. However, GNP retention in the tumour 24 h post-treatment was only 20%. This is attributed to the natural clearance of GNPs from the circulation after 24 h, before free DTX or the LNP_DTX-P_ had the opportunity to exert their full effect. This is supported by the flow cytometry cell cycle data for the 8 h time point, which show that most of the tumour cells were in the G1 phase (Figure 4B). As the DTX exposure time increased, the effects on the MTs increased the GNP accumulation within the tumour, as demonstrated by the synchronization of a larger number of tumour cells in the G2/M phases after 24–48 h for both the LNP_DTX-P_ and the free DTX (Figure 4B). This resulted in a similar number of GNPs at the 48 h and 24 h time points for both the free-DTX- and LNP_DTX-P_-treated tumours. Furthermore, the LNP_DTX-P_-treated samples had a higher population of cells in the G2/M phases compared to the free-DTX-treated samples. This was likely the result of LNPs bringing more DTX to the tumour cells compared to free DTX. On the other hand, the untreated tumours showed a further decrease in the number of accumulated GNPs due to the exocytosis process and the lack of GNPs circulating in the blood after 48 h. These results are further supported by darkfield images of the tumour tissues of the untreated samples, free-DTX-treated samples, and LNP_DTX-1_-treated samples (Figure 4C). Additional darkfield images of the LNP_DTX-2_-treated samples are provided in the supplementary file (Figure S3). An immediate visual increase in the number of GNPs was observed when comparing the tumour samples treated with free DTX or LNP_DTX-P_ to the untreated samples for the different time points, which agreed with our quantification data. The results showed no significant difference between the two LNP_DTX-P_ used compared to free DTX, consistent with the in vitro results. Furthermore, a clear increase in DTX-induced cell damage was observed for the two LNP_DTX-P_ treatments and the free DTX treatment compared to the control sample, as demonstrated in the hematoxylin and eosin (H&E) stained 8 h, 24 h, and 48 h post-treatment tumours (Figure 4D). The organ H&E images seen in the Supplementary Materials (Figure S4A–C) do not seem to show any observable effects with the given dose of DTX for either the LNP_DTX-P_ or the free DTX.

Despite its toxicity, free DTX has already shown remarkable radiosensitization effects in several clinical trials [44,45,46,47,48,49]. Hence, our results are very promising, as they show the potential of using the LNP_DTX-1_ and LNP_DTX-2_ formulations instead of free DTX as potential radiosensitizers, as they deliver similar synchronizations of cancer cells in the radiosensitive G2/M phases and similar GNP uptake and retention. This is very important when considering combined chemoradiation and nanotherapy treatments since DTX is administered weekly to patients. With the synchronization of cells in the G2/M phases for at least 48 h following treatment and with the trapping of GNPs in the tumour, a 5-day-a-week fractionated radiotherapy treatment regime would work synergistically.

**Figure 5:**
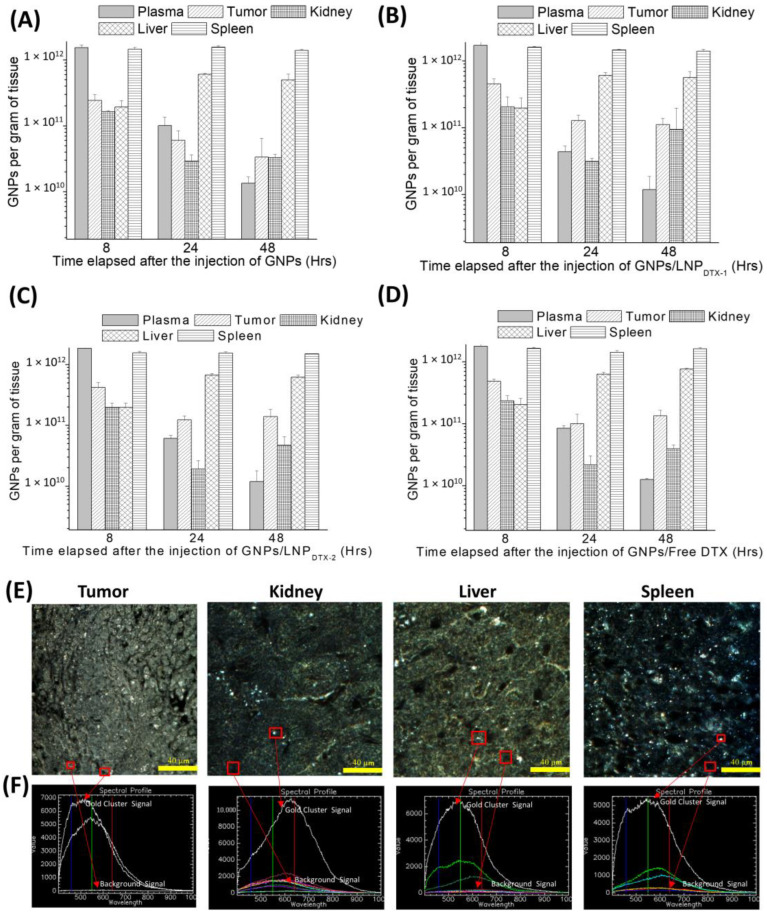
Gold nanoparticle (GNPs) uptake in in vivo tumour tissues and organs. (**A**–**D**) GNP amounts per gram of tissue for untreated mice, mice treated with LNP_DTX-1_, mice treated with LNP_DTX-2_, and mice treated with free DTX, respectively. (**E**) Darkfield images of 4 µm sections of tumour tissue, kidney, liver, and spleen 24 h after LNP_DTX-1_ treatment. (**F**) Hyperspectral spectra of GNPs within their respective tissues. Scale bar: 40 µm.

The GNP contents, normalized to GNPs per gram of tissue, in the control organs (Figure 5A), organs treated with LNP_DTX-1_ (Figure 5B), organs treated with LNP_DTX-2_ (Figure 5C), and organs treated with free DTX (Figure 5D) are shown in Figure 5. Despite the clear increase in GNP uptake and retention in free-DTX- and LNP_DTX-P_-treated tumours, neither free DTX nor the LNP_DTX-P_ significantly increased the number of GNPs in normal organs. This implies that there was an increase in the accumulation of GNPs in tumours relative to other organs over 48 h due to DTX tumour targeting and the EPR effect. The results also show that GNPs had longer retention in tumours compared to GNPs in the blood in circulation. This is supported by recent pharmacokinetic studies that showed similar accumulations of GNPs in treated tumours compared to healthy organs [50]. This is very important when considering radiotherapy, as it shows that both DTX and GNPs will remain within the tumour, thus allowing for a synergistic radiosensitization effect up to at least 48 h. Darkfield images of the organs treated with LNP_DTX-1_ after 24 h show no signs of damage in these organs compared to the tumours (Figure 5E). Hyperspectral images were used for GNP verification (Figure 5F). Additional darkfield images of untreated organs, organs treated with free DTX, and organs treated with LNP_DTX-2_ 24 h post-treatment are presented in the Supplementary Materials (Figure S5).

## 4. Conclusions

The use of nanotechnology in cancer treatment has the potential to solve many of the problems of conventional cancer therapeutics. The radiosensitization effect of GNPs can enhance the toxicity to cancer tissue without negatively impacting normal tissue. Moreover, the addition of DTX to GNPs results in an increase in the accumulation of GNPs within tumour cells, which allows for a synergetic effect that can further enhance the cancer therapeutic effect, albeit with additional normal tissue toxicity. In this paper, we measured the uptake and retention of GNPs in vitro and in vivo, using the pancreatic cancer cell line MIA PaCa-2 following treatment with free DTX vs. a DTX prodrug encapsulated in LNPs (LNP_DTX-P_). Both in vitro and in vivo, the addition of free DTX and LNP_DTX-P_ displayed significant increases in GNP uptake relative to control samples, with LNP_DTX-P_ displaying similar cancer toxicity when compared to free DTX. Moreover, the quantitative and qualitative results did not show any significant difference between the two LNP formulas that we used, LNP_DTX-1_ and LNP_DTX-2_. This was expected, considering the IC-50 doses of both formulas show roughly the same amount of active drug in them. These results are very promising, as LNP_DTX-P_ have superior targeting of tumour tissues compared to free DTX. Because of their minimal toxicity to normal tissues, we expect GNPs and LNP_DTX-P_ to be ideal radiosensitization candidates in radiotherapy, with promising synergistic therapeutic outcomes that will improve patients’ quality of life. This will be explored in depth in our future experiments.

## Data Availability

The datasets used and/or analyzed during the current study are available from the corresponding author upon reasonable request.

## Supplementary Materials

The following supporting information can be downloaded at: https://www.mdpi.com/article/10.3390/cancers14246137/s1, Figure S1: Synthetic scheme for DTX prodrug, Figure S2: Characterization of gold nanoparticles (GNPs) and lipid nanoparticles (LNPs), Figure S3: Darkfield images of 4 µm sections of tumour tissues treated with LNPDTX-2. Scale bar: 40 µm, Figure S4: (A): Hematoxylin and eosin stained sections of kidneys 0 h, 24 h, and 48 h after dosing with the drugs and GNPs. Scale bar: 80 µm, (B): Hematoxylin and eosin stained sections of liver 0 h, 24 h, and 48 h after dosing with the drugs and GNPs. Scale bar: 80 µm, (C): Hematoxylin and eosin stained sections of spleen 0 h, 24 h, and 48 h after dosing with the drugs and GNPs. Scale bar: 80 µm; Figure S5: Darkfield images of 4 µm sections of kidney, liver, and spleen for control samples, LNPDTX-2-treated samples, and free-DTX-treated samples 24 h post-treatment, respectively. Scale bar: 40.
