# Supplementary material for: Lipid-Nanoparticle-Mediated Delivery of Docetaxel Prodrug for Exploiting Full Potential of Gold Nanoparticles in the Treatment of Pancreatic Cancer
Source: Cancers (Basel). 2022 Dec 13;14(24):6137. doi: 10.3390/cancers14246137 (PMC9776798; doi:10.3390/cancers14246137)
Supplement: Supplementary file 1 [file cancers-14-06137-s001.zip › cancers-2043801-supplementary.pdf]

cancers

MDPI

Supplementary Materials: Lipid-Nanoparticle-Mediated Delivery of Docetaxel Prodrug for Exploiting Full Potential of Gold Nanoparticles in the Treatment of Pancreatic Cancer

Abdulaziz Alhussan, Nolan Jackson, Sarah Eaton, Nancy Dos Santos, Ingrid Barta, Josh Zaifman, Sam Chen, Yuen Yi C. Tam, Sunil Krishnan and Devika B. Chithrani

1. Supplementary Section S1: DTX Prodrug Synthesis

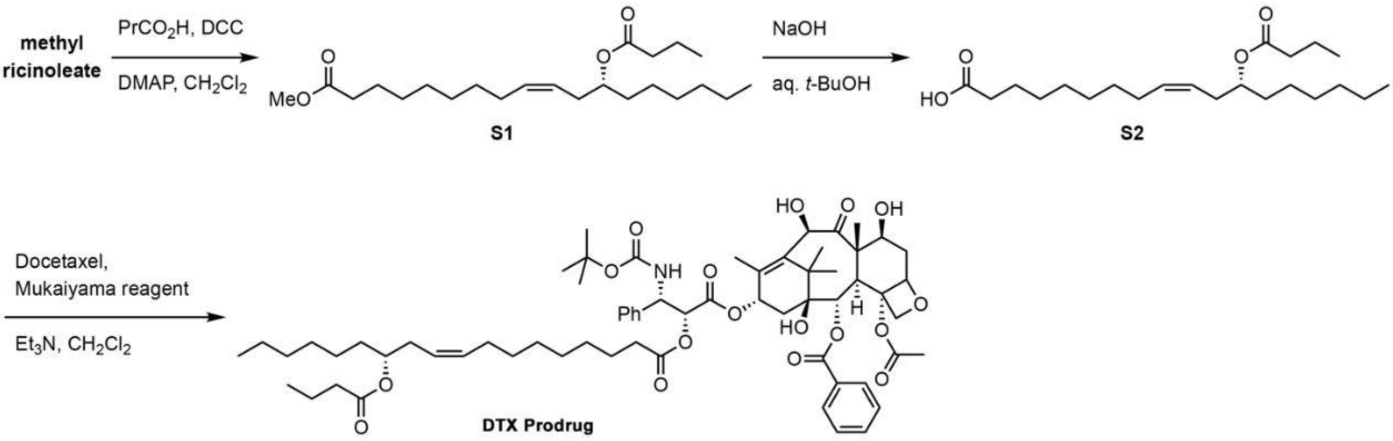

Figure S1. Synthetic scheme for Docetaxel (DTX) prodrug.

1.1. Methyl (R,Z)-12-(butyryloxy)octadec-9-enoate (S1)

Butanoic acid (1.22 mL, 13.2 mmol) was added to an ice-cold CH_2Cl_2 (24 mL) solution of DCC (2.72 g, 13.2 mmol) in a round bottom flask under argon and the ice bath was removed. After 20 min, the ice bath was replaced, methyl ricinoleate (3.75 g, 12.0 mmol) and DMAP (2.20 g, 18.0 mmol) were added and the resultant was allowed to warm up over 14 h. The reaction mixture was diluted with hexanes, filtered through Celite® and concentrated on a rotary evaporator. The crude was resuspended in hexanes, washed with aqueous 1 M HCl (2 × 50 mL), aqueous 1 M NaOH (2 × 50 mL), H_2O (1 × 50 mL), brine, dried over Na_2SO_4 and concentrated on a rotary evaporator to afford a clear, colorless oil as desired diester S1 (4.31 g, 94% yield), which was used without further purification.

1.2.(R,Z)-12-(Butyryloxy)octadec-9-enoic acid (S2)

Aqueous 2.0 M KOH (5.35 mL, 10.7 mmol) was added to a room temperature *t*-BuOH (35 mL) solution of methyl ester S1 (4.30 g, 11.2 mmol) in a round bottom flask under argon and stirred for 14 h. The reaction mixture was adjusted to pH < 2 by addition of aqueous 6 M HCl and extracted with hexanes (3 × 50 mL). The combined organic extracts were washed with aqueous 1 M HCl (1 × 50 mL), brine, dried over Na_2SO_4 and concentrated on a rotary evaporator. The crude was purified by flash column chromatography (95:5:0→80:15:5→60:30:10 hexanes-EtOAc-MeOH) to afford a clear, colorless oil as desired carboxylic acid S2 (3.02 g, 77% yield based on KOH).

1.3. Docetaxel Prodrug

Triethylamine (0.17 mL, 1.25 mmol), followed by Mukaiyama reagent (166 mg, 0.65 mmol), was added to a CH_2Cl_2 (5 mL) suspension of docetaxel (404 mg, 0.50 mmol) and

carboxylic acid S2 (0.55, 1.10 mmol) and the resultant stirred for 14 h. The reaction mixture was concentrated on a rotary evaporator and purified by flash column chromatography (80:20→50:50 hexanes-EtOAc) to afford a clear, colorless oil as desired docetaxel prodrug (262 mg, 45% yield). Analytic TLC was carried out with Merck silica gel 60 plates with fluorescent indicator; spots were visualized with UV light, iodine vapor or permanganate stain. The structure and purity of all final compounds were ascertained by NMR spectroscopy (^{1}H at 300 MHz, ^{13}C at 75 MHz, recorded at ambient temperature in $CDCl_3$).

2. Supplementary Section S2: GNPs and LNPs Characterization

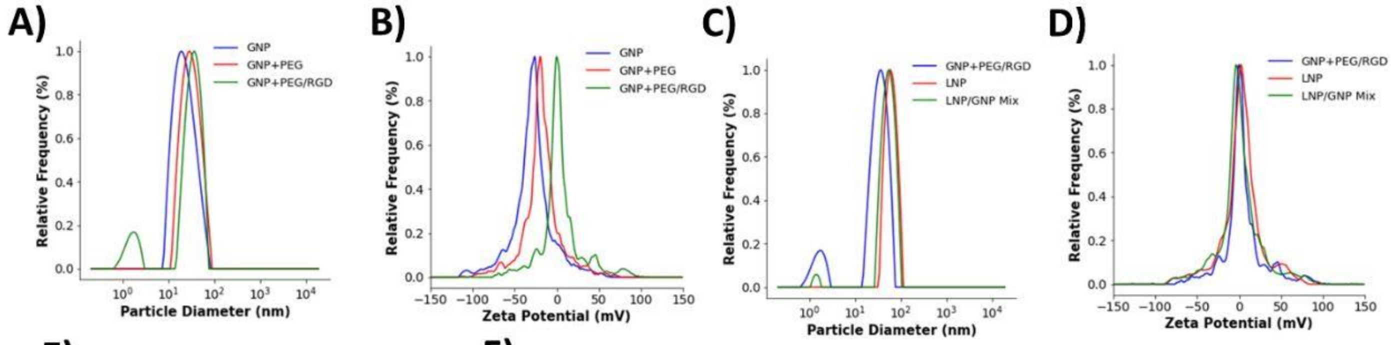

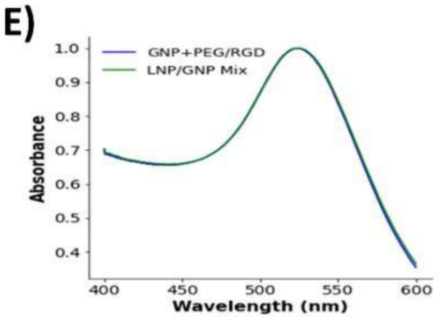

F)

Ligands	Peak Wavelength (nm)	Hydrodynamic Diameter (nm)	Zeta Potential (mV)
GNP	519.45 ± 0.05	15.11 ± 0.92	-19.2 ± 3.6
GNP + PEG	520.40 ± 0.05	22.43 ± 1.34	-3.1 ± 0.73
GNP + PEG/RGD	525.45 ± 0.05	27.0 ± 2.41	-0.9 ± 0.9
LNP	N/A	62.5 ± 5.19	-0.8 ± 1.2
LNP/GNP Mix	525.15 ± 0.05	53.0 ± 3.65	-1.1 ± 1.0

Figure S2. Characterization of gold nanoparticles (GNPs) and lipid nanoparticles (LNPs). (**A-B**) Hydrodynamic diameter and ζ-potential measurements of pure GNPs, GNP$_{PEG}$, and GNP$_{PEG\text{-}RGD}$, respectively. (**C-D**) Hydrodynamic diameter and ζ-potential measurements of GNP$_{PEG\text{-}RGD}$, LNP$_{DTX\text{-}P}$, and LNP$_{DTX\text{-}P}$/ GNP$_{PEG\text{-}RGD}$, respectively. **(E)** UV–Visible absorption spectra of GNPPEG-RGD and LNP$_{DTX\text{-}P}$/ GNP$_{PEG\text{-}RGD}$. **(F)** Summary of peak absorption wavelength, hydrodynamic diameter, and mean ζ-potential for pure GNPs, GNP$_{PEG}$, GNP$_{PEG\text{-}RGD}$, LNP$_{DTX}$ and LNP$_{DTX\text{-}P}$/ GNP$_{PEG\text{-}RGD}$.

3. Supplementary Section S3: Darkfield Tumour Tissue

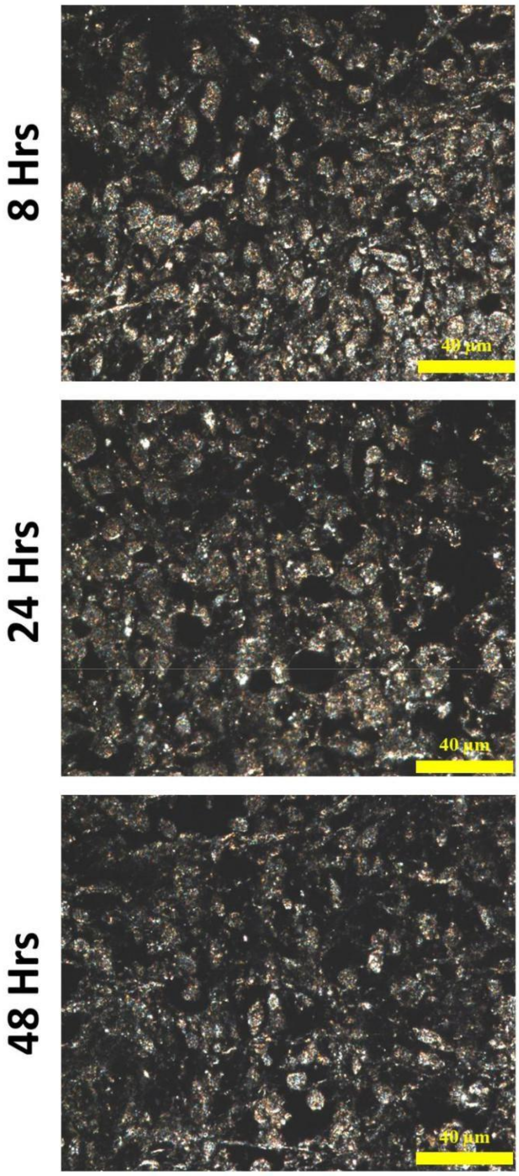

Figure S3. Darkfield images of 4 µm sections of tumour tissues treated with LNP_{DTX-2}. Scale bar: 40 µm.

4. Supplementary Section S4: Images of Organs H&E

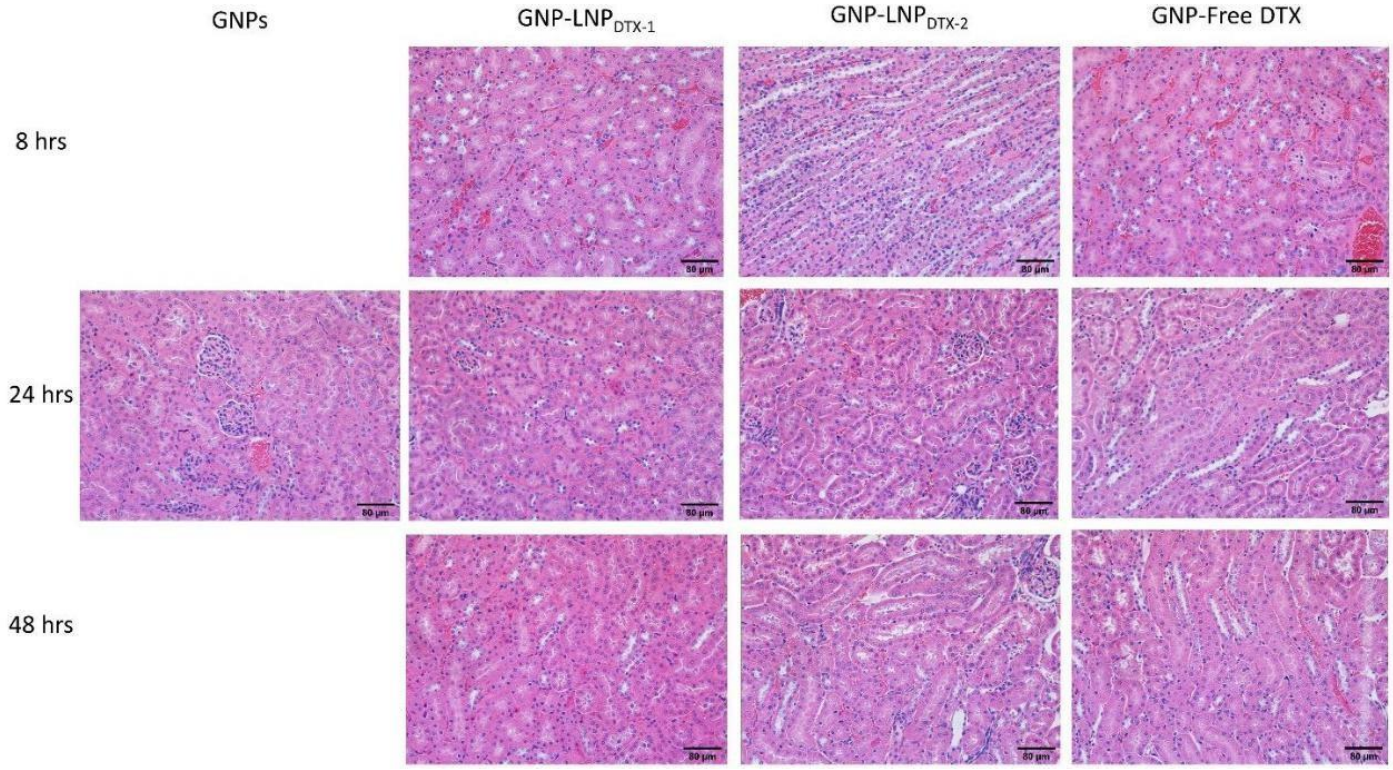

Figure S4. A. Hematoxylin and eosin-stained sections of kidneys 0 h, 24 h, and 48 h after dosing with the drugs and GNPs. Scale bar: 80 µm.

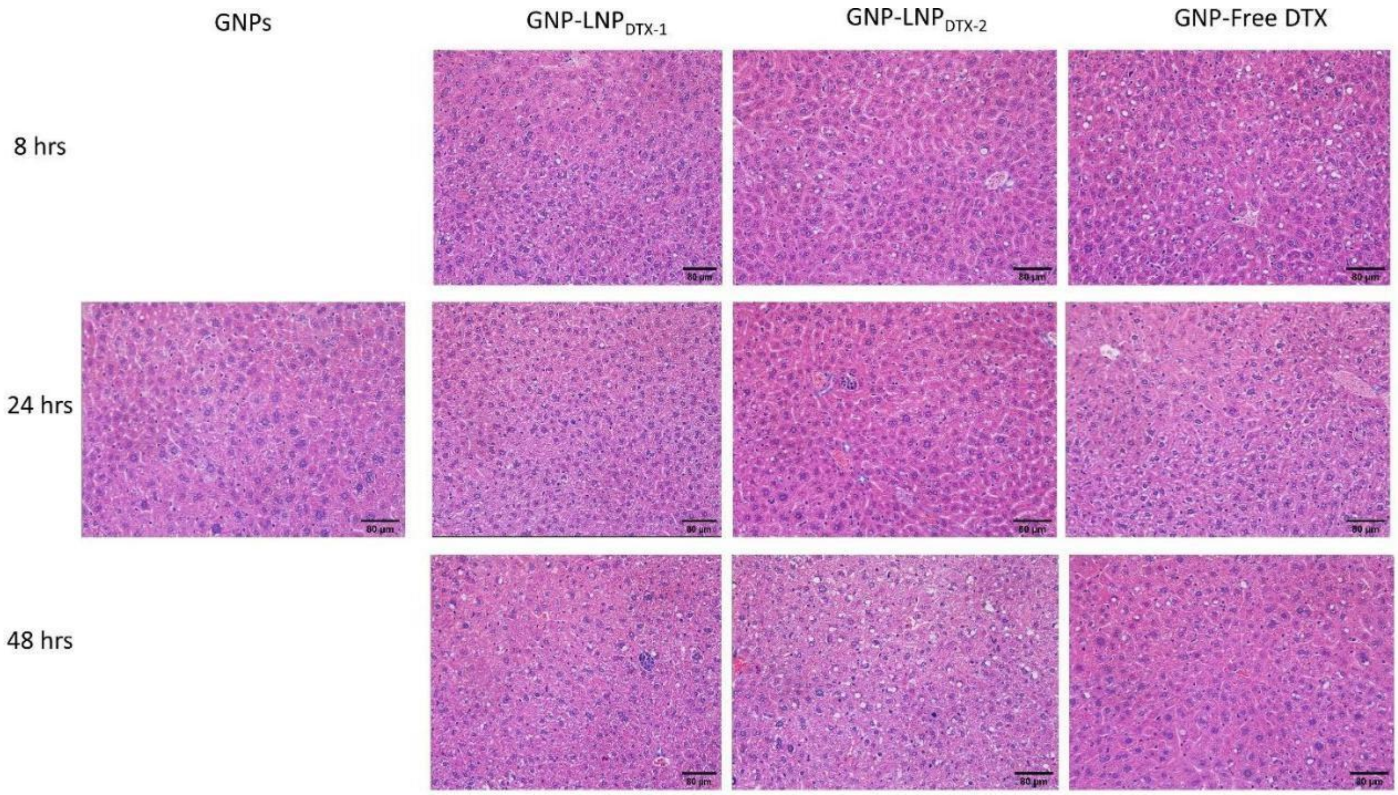

Figure S4. B. Hematoxylin and eosin-stained sections of liver 0 h, 24 h, and 48 h after dosing with the drugs and GNPs. Scale bar: 80 µm.

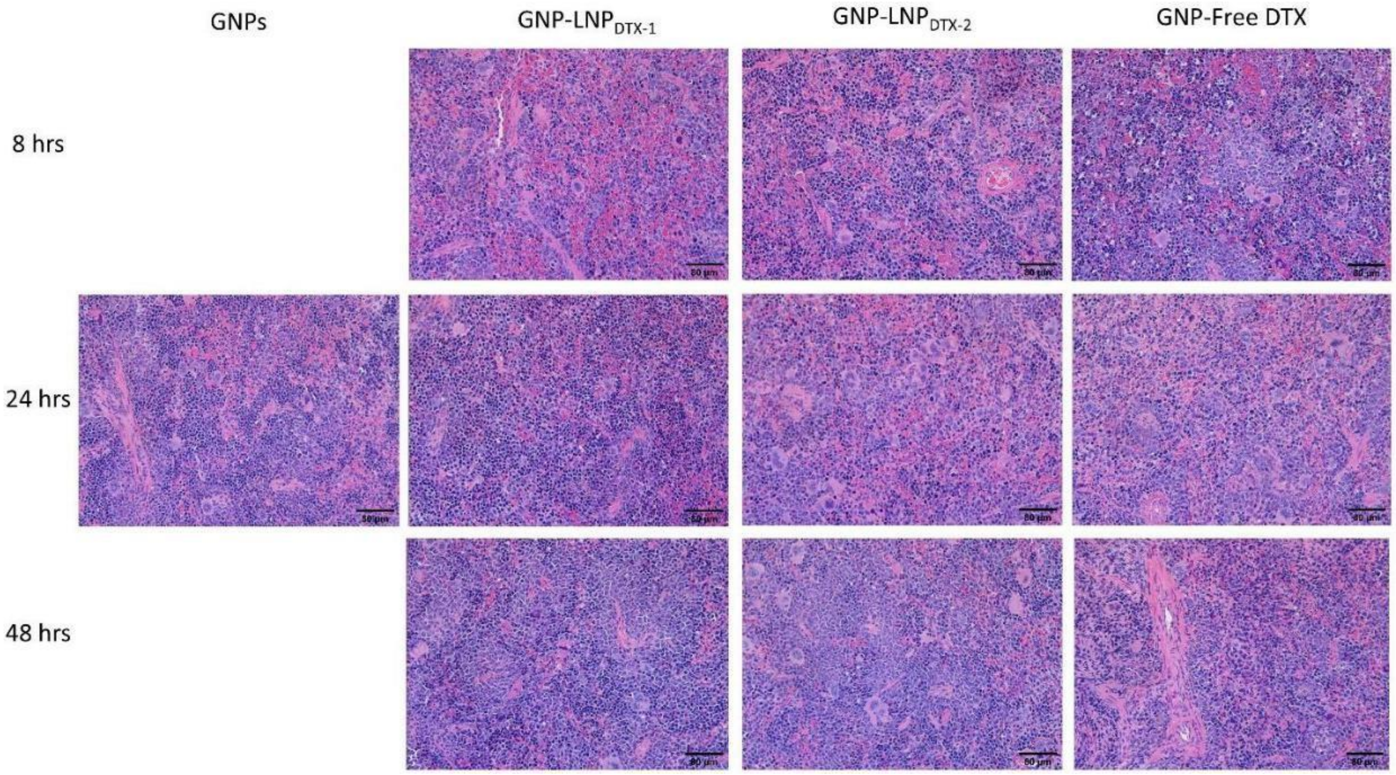

Figure S4. C. Hematoxylin and eosin-stained sections of spleen 0 h, 24 h, and 48 h after dosing with the drugs and GNPs. Scale bar: 80 µm.

5. Supplementary Section S5: Organs Darkfield Images

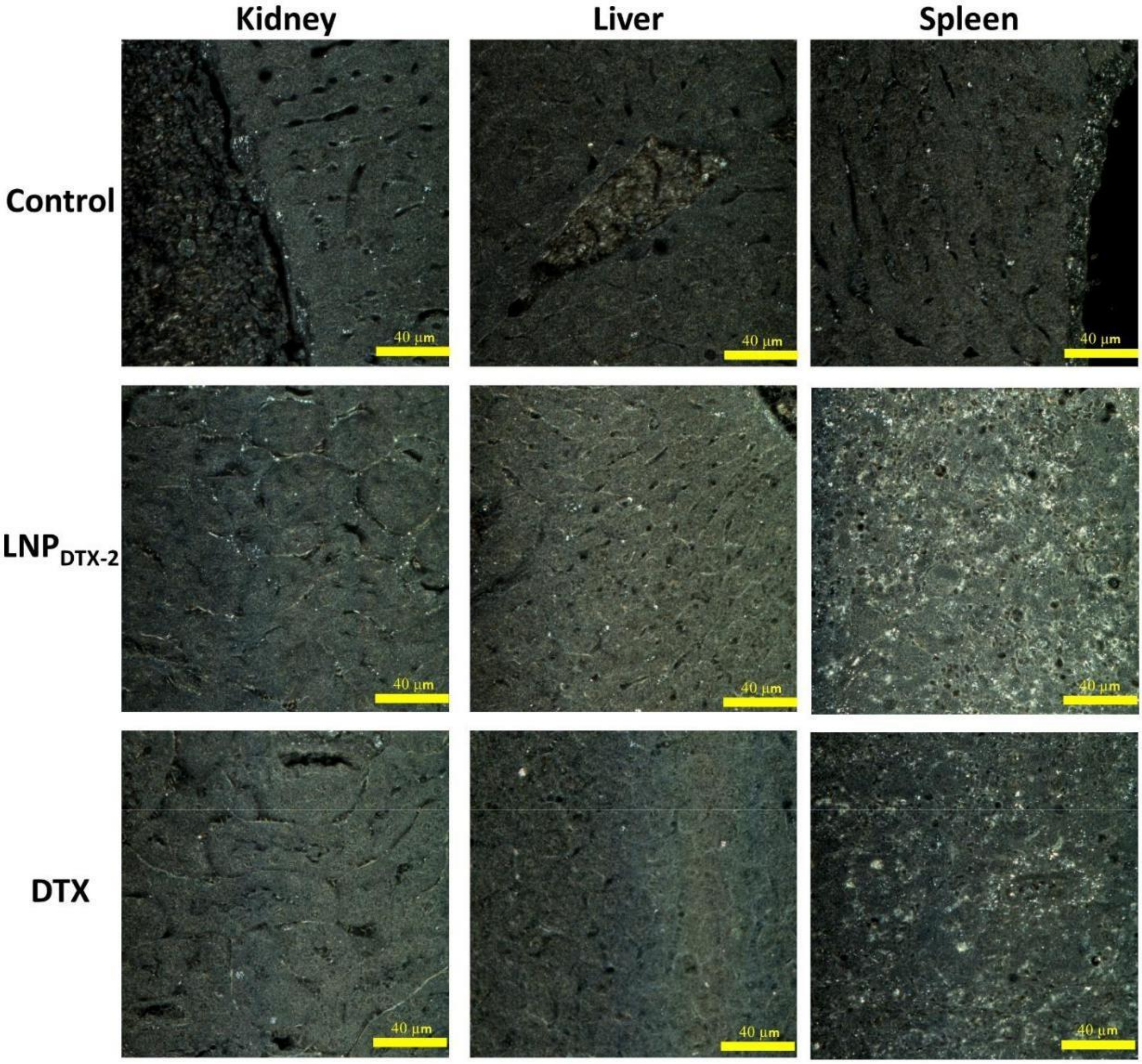

Figure S5. Darkfield images of 4 µm sections of kidney, liver, and spleen, for control samples, LNP$_{DTX-2}$ treated samples, and free DTX treated samples, 24 h post treatment, respectively. Scale bar: 40 µm.
